# Design, Synthesis and Preliminary Pharmacological Evaluation of New Non-Steroidal Anti-Inflammatory Agents Having a 4-(Methylsulfonyl) Aniline Pharmacophore 

**DOI:** 10.3390/molecules17021751

**Published:** 2012-02-10

**Authors:** Monther Faisel Mahdi, Mohamed Hassan Mohammed, Akeel Abdul Kadhum Jassim

**Affiliations:** 1 Department of Pharmaceutical Chemistry, College of Pharmacy, University of Baghdad, Baghdad, 10047, Iraq; 2 Department of Pharmaceutical Chemistry, College of Pharmacy, University of Kufa, Najaf, 54001, Iraq

**Keywords:** anti-inflammatory, paw edema, NSAIDs, naproxen, indomethacin, mefanamic acid, diclofenac

## Abstract

A series of 4-(methylsulfonyl)aniline derivatives were synthesized in order to obtain new compounds as a potential anti-inflammatory agents with expected selectivity against COX-2 enzyme. *In vivo *acute anti-inflammatory activity of the final compounds **11**–**14** was evaluated in rat using an egg-white induced edema model of inflammation in a dose equivalent to 3 mg/Kg of diclofenac sodium. All tested compounds produced significant reduction of paw edema with respect to the effect of propylene glycol 50% v/v (control group). Moreover, the activity of compounds **11** and **14** was significantly higher than that of diclofenac sodium (at 3 mg/Kg) in the 120–300 minute time interval, while compound **12** expressed a comparable effect to that of diclofenac sodium in the 60–240 minute time interval time, and compound **13** showed a comparable effect to that of diclofenac sodium at all experimental times. The result of this study indicates that the incorporation of the 4-(methylsulfonyl)aniline pharamacophore into naproxen, indomethacine, diclofenac and mefanamic acid maintained their anti-inflammatory activity and may increase selectivity towards the COX-2 enzyme which will be confirmed in the future by assessing COX-2: COX-1 inhibitory ratios using a whole blood assay.

## 1. Introduction

**Figure 1:**
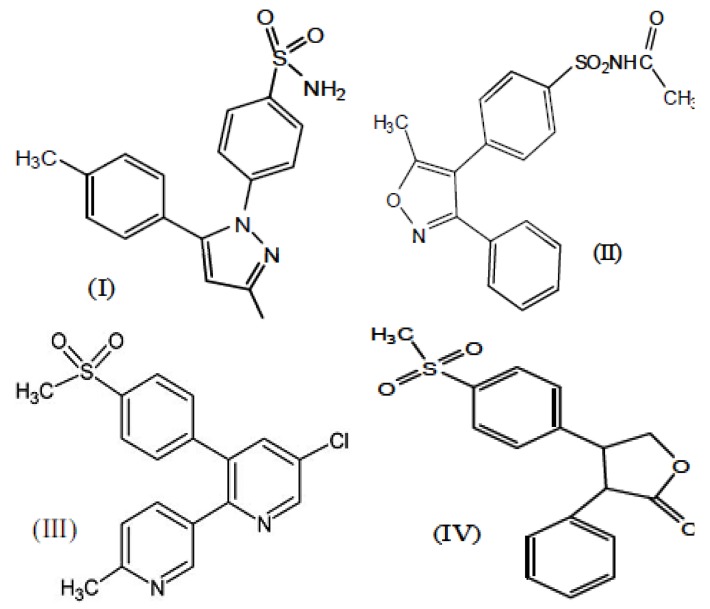
Common selected COX-2 inhibitors.

Non-steroidal anti-inflammatory drugs (NSAIDs) are used to treat a wide variety of illnesses and diseases, including inflammation [1], cancers [2], diabetes [3] (insulin-resistant and related metabolic syndrome); and diseases of the peripheral and central nervous system, e.g., Alzheimer’s and Parkinson’s [4]. This versatility is attributed to a wide variety of effects of these drugs on cell function. The anti-inflammatory effect of NSAIDs arises from their ability to inhibiting cyclooxygenase (COX) enzyme [1]. The COX enzymes catalyze the bis-dioxygenation and subsequent reduction of arachidonic acid (AA) to prostaglandin (PG) G2 and PGH2 [5]. Three different COX enzymes exist, known as COX-1, COX-2 and COX-3, COX-1 is a constitutive isoform found in most normal cells and tissues [6]. It is stimulated by growth factor and hormones and it has been called the housekeeping enzyme [7]. The COX-1 plays fundamental roles in the generation of PGs in homoeostasis [8], and several other physiological functions including gastric protection and control of renal blood flow [9]. Moreover, COX-1 regulates the physiological process of platelet aggregation [7]. COX-2 is the readily inducible form of the enzyme and it is commonly associated with several pathological conditions in the heart [10], spinal cord [11], vascular endothelium, brain, kidney, bone and female reproductive system and is also involved in certain physiological processes [12,13]. However, its expression at other sites is increased during states of inflammation or, experimentally, in response to mitogenic stimuli. As an example, growth factors, phorbol esters, and interleukin-1 all stimulate the expression of COX-2 in fibroblasts, and it is also induced by inflammatory stimuli such as bacterial endotoxins and cytokines [14,15]. COX-3 is a recently identified splice variant/isoenzyme of COX-1 and, more suitably, may have been named COX-1b. In humans COX-3 mRNA is found in highest concentrations in the brain and heart [16]. The importance of COX-3 is that it could explain the pharmacological actions of paracetamol and other antipyretic analgesic drugs which are weak inhibitors of COX-1 and COX-2, but penetrate easily into the central nervous system [17]. Selective COX-2 inhibitors differ from traditional NSAIDs in two major ways, Coxibs are less likely to result in NSAID-induced gastropathy, and they do not inhibit platelet function [18]. As a result, selective COX-2 inhibitors elicit less clinically significant GI damage and bleeding than conventional NSAIDs [19]. The cardiovascular toxicity of selective COX-2 inhibitors is possibly a consequence of the inhibition of the synthesis of prostacyclin (PGI2), which has anti-thrombotic properties, while sparing the synthesis of thromboxane A2 (TXA2), a pro-thrombotic substance, PGE2 is the PG primarily associated with inflammation. Therefore, selective inhibition of PGE2 synthesis could be a rational approach for reducing inflammation without producing the cardiovascular and GI toxicity associated with NSAIDs [20]. In 1991 when COX-2 was discovered, scientists started focusing on selective COX-2 inhibitors. *In vitro* recombinant enzyme assays provided a powerful means for assessing COX selectivity and potency and led to the discovery and clinical development of the rationally designed COX-2 selective inhibitors, celecoxib (**I**) and parecoxib (**II**) with a sulfonamide substituent in the *para*-position on one of the aryl rings while etoricoxib (**III**) and rofecoxib (**IV**) have a methylsulfone, as shown in Figure 1 [21]. In place of the carboxyl group of the non-steroidal anti-inflammatory acids, the sulfur-containing phenyl ring of these drugs (and maybe our analogues) binds into the side pocket of the cyclooxygenase catalytic channel of COX-2, but interacts weakly with the active site of COX-1 [22]. 

In the view of this background, the present study was conducted to design, synthesize and preliminarily evaluate some new non-steroidal anti-inflammatory agents with expected selectivity toward COX-2 enzyme. There is evidence to suggest that COX-2 selective inhibitors may inhibit COX-1 and induce GI irritation or ulceration with long term use or at higher doses [23,24]. Preclinical cardiovascular and renal liabilities of at least some COX-2 selective inhibitors have also been reported [25]. Thus, there is still a need for new, selective COX-2 inhibitors with an improved safety profile.

## 2. Results and Discussion

Many irritant agents have been used in the paw-edema method like dextran, egg-white and carrageenan solution. The paw edema induced by carrageenan has been extensively studied in the assessment of the anti-inflammatory action of steroidal and non-steroidal drugs involving several chemical mediators such as histamine, serotonin, bradykinin and prostaglandins [26]; the intraplantar injection of egg-white into rat hind paw induces a progressive edema. To assess the validity of the method (paw edema) used for the evaluation of newly synthesized anti-inflammatory compounds, diclofenac sodium was used as a reference compound of known anti-inflammatory activity profile.

**Figure 2:**
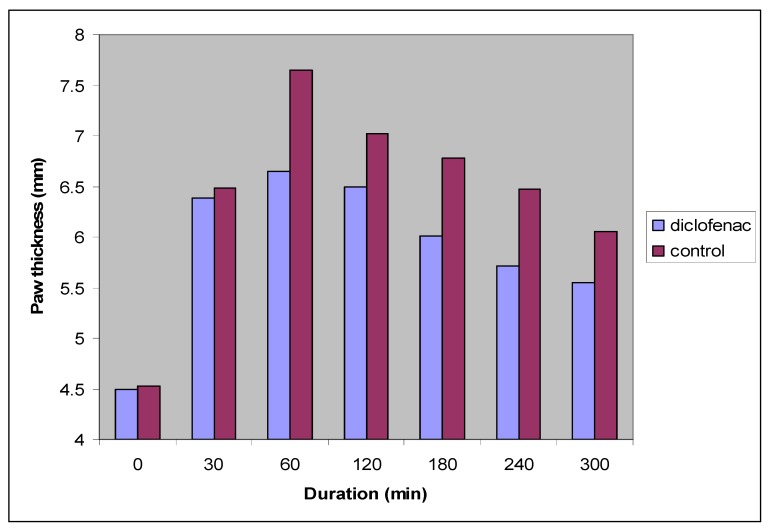
Effect of diclofenac sodium (reference), and propylene glycol (control) on egg-white induced paw edema in rats. Time (30) is the time of egg-white injection.

Table 1 shows the effect of diclofenac sodium (reference) and propylene glycol (control) on egg-white induced paw edema in rats. The differences in paw thickness readings among control and diclofenac sodium groups indicates that the method used in this study (paw edema) is a valid method and can effectively be used for the assessment of the anti-inflammatory effect of the newly synthesized compounds as shown in Figure 2.

**Table 1:** Effect of diclofenac sodium (reference) and propylene glycol (control) on egg-white induced paw edema in rats.

	Time (min)	Control (n = 6)	Diclofenac sodium (n = 6)
**Paw thickness (mm)**	0	4.53 ± 0.19	4.50 ± 0.08
	30	6.48 ± 0.11	6.38 ± 0.14
	60	7.65 ± 0.16	6.65 ± 0.18 *
	120	7.02 ± 0.18	6.49 ± 0.04 *
	180	6.78 ± 0.04	6.01 ± 0.11 *
	240	6.47 ± 0.11	5.71 ± 0.12 *
	300	6.06 ± 0.03	5.55 ± 0.03 *

Data are expressed in mm paw thickness as mean ± SEM; n = number of animals; Time (0) is the time of i.p. injection of diclofenac sodium and propylene glycol; Time (30) is the time of injection of egg-white (induction of paw edema); * significantly different compared to control (*p* < 0.05).

**Figure 3:**
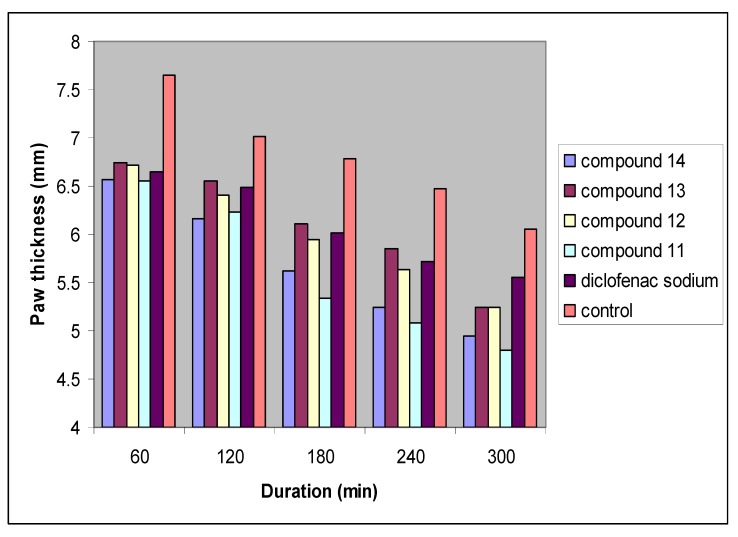
Effect of diclofenac sodium, propylene glycol, compounds 11, 12, 13 and 14 on egg-white induced paw edema in rats. Results are expressed as mean ± SEM (n = 6 for each group). Time (30) is the time of egg-white injection.

Table 2 shows the effect of the tested compounds **11**–**14** with respect to control and reference group (diclofenac sodium). All tested compounds effectively limited the increase in paw edema, the effect of all tested compounds started at 60 minutes (significantly different compared to control). However, the effect of all tested compounds continued till the end of the experiments with statistically significant (*P* > 0.05) reduction in paw edema, as shown in Figure 3.

**Table 2:** Effect of Control, Diclofenac and Compounds **11**–**14** on egg-white induced paw edema in rats.

Treatment groups							
	Time (min)	Control (n = 6)	Diclofenac sodium (n = 6)	Compound 11 (n = 6)	Compound 12 (n = 6)	Compound 13 (n = 6)	Compound 14 (n = 6)
**Paw thickness (mm)**	0	4.53 ± 0.19	4.50 ± 0.08	4.55 ± 0.11	4.49 ± 0.06	4.45 ± 0.08	4.43 ± 0.06 *
	30	6.48 ± 0.11	6.38 ± 0.14	6.33 ± 0.04	6.40 ± 0.11	6.39 ± 0.06	6.35 ± 0.14
	60	7.65 ± 0.16	6.65 ± 0.18 *	6.55 ± 0.13 *	6.72 ± 0.12 *	6.75 ± 0.04 *	6.57 ± 0.03 *
	120	7.02 ± 0.18	6.49 ± 0.04 *^a^	6.23 ± 0.06 *^b^	6.41 ± 0.05 *^a^	6.56 ± 0.06 *^a^	6.16 ± 0.18 *^b^
	180	6.78 ± 0.04	6.01 ± 0.11 *^a^	5.34 ± 0.04 *^b^	5.95 ± 0.03 *^a^	6.11 ± 0.08 *^a^	5.62 ± 0.04 *^c^
	240	6.47 ± 0.11	5.71 ± 0.12 *^a^	5.08 ± 0.10 *^b^	5.64 ± 0.04 *^a^	5.85 ± 0.16 *^a^	5.24 ± 0.05 *^b^
	300	6.06 ± 0.03	5.55 ± 0.03 *^a^	4.80 ± 0.02 *^b^	5.25 ± 0.06 *^c^	5.52 ± 0.12 *^a^	4.94 ± 0.12 *^b^

Non-identical superscripts (a & b) among different tested compounds are considered significantly different (*p* < 0.05); * significantly different compared to diclofenac (*p* < 0.05).

The comparison between the tested compounds and diclofenac sodium shows that at time 0–60 minutes there are no differences with diclofenac sodium between groups; however at the interval time 120–300 minute, compounds **11** and **14** show significantly higher effects than diclofenac sodium, while compound **12** expressed a comparable effect to that of diclofenac sodium at the interval time 60–240 minute, and compound **13** showed a comparable effect to that of diclofenac sodium for all the experimental times.

## 3. Experimental

### 3.1. General

All reagents and anhydrous solvents were of analar type and generally used as received from the commercial supplierxs (Merck, Germany, Reidel-De Haen, Germany, Sigma-Aldrich, Germany and BDH, England). Naproxen, indomethacin and mefanamic acid was supplied by the SDI Company, Iraq. Melting points were determined by capillary method on Bamstead/Electrothermal 9100 an Electric melting point apparatus (England) and ascending thin layer chromatography (TLC) to check the purity and progress of reactions was run on DC-Kartan SI alumina 0.2 mm plates. The identification of compounds was done using a U.V. detector and the chromatograms were eluted with THF-ether-cyclohexane (4:4:2). IR spectra were recorded on a FTIR-spectrophotometer Shimadzu as KBr disks. CHNS microanalysis was done using a Euro EA 3000 elemental analyzer (Italy). 

**Scheme 1:**
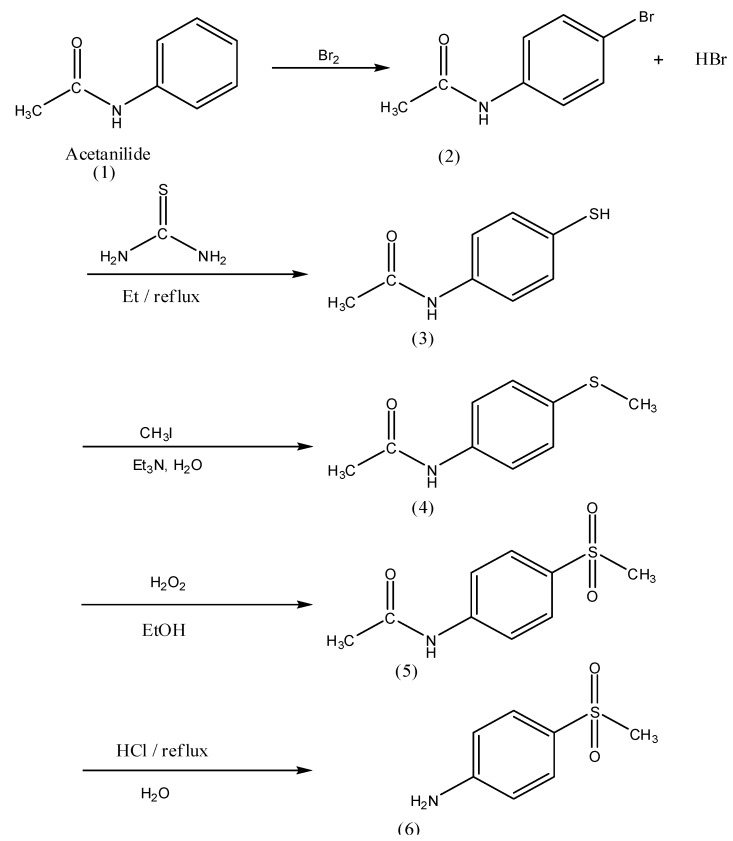
Synthesis of 4-(methylsulfonyl)aniline (**6**).

**Scheme 2:**
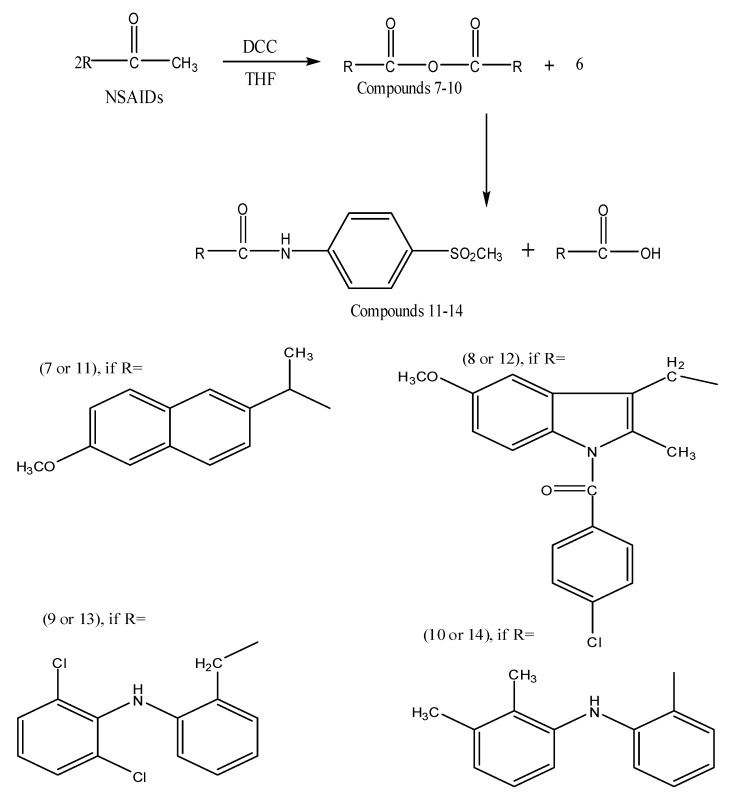
Synthesis of target compounds **11–14**.

The general routes outlined in Scheme 1 and Scheme 2 were used to synthesize all compounds described here. As shown in Scheme 1, 4-(methylsulfonyl)aniline (**6**) was synthesized starting from acetanilide.

### 3.2. Synthesis of N-(4-Bromophenyl)acetamide (***2***)

Finely powdered acetanilide (3.38 g, 25 mmol) was dissolved in glacial acetic acid (11.25 mL) in a conical flask. In another flask bromine (1.32 mL, 25 mmol) was mixed with glacial acetic acid (6.22 mL) and the solution transfered to a separatory funnel. The bromine solution was added slowly to the first flask with constant shaking to ensure thorough mixing. The flask was left standing in cold water. When all the bromine has been added, the solution will have an orange color and a part of the reaction product may crystallize out. The final reaction mixture was allowed to stand at room temperature for 30 min with occasional shaking. The reaction product was poured into a flask containing distilled water (100 mL) and the mixture was stirred well. The crystalline precipitate was filtered with suction on a Buchner funnel, washed thoroughly with cold distilled water and pressed as dry as possible with a wide glass stopper. Recrystallization from dilute ethanol [27] gave the title compound as a white powder (80% yield); m.p. 166–167 °C (lit. [27] 167–169 °C); R_f_ = 0.77; IR (cm^−1^): 3,306 (N-H) of secondary amide, 1,672 (C=O) of secondary amide, 1,600, 1,535 and 1,485 (aromatic), 1,311 (C-N) of secondary aromatic amide, 688 (C-Br).

### 3.3. Synthesis of N-(4-Mercaptophenyl)acetamide (***3***)

The intermediate **2** (2.13 g,10 mmol) was dissolved in absolute 99% ethanol (60 mL) and refluxed under 50 °C with an excess amount of thiourea (2.28 g, 30 mmol) overnight with stirring. After cooling the reaction mixture to room temperature the solvent was evaporated and the residue then hydrolyzed in 2 N NaOH under stirring with gentle heating, then acidified with a mixture consisting of conc. HCl (3 mL) and water (10 mL). The formed solid was filtered, washed with water and recrystallized from ethanol [28] to give compound **3** as a yellow powder (33% yield); m.p. 151–153 °C (lit. [as stated by the manufacturer, Shanghai Yancui Import & Export Co., Ltd.] 149–154 °C); R_f_ = 0.71. IR (cm^−1^): 3,296 (N-H) of secondary amide, 2,548 (S-H) of thiol, 1,662 (C=O) of secondary amide, 1,599, 1,537 and 1,491 (aromatic), 1,317 (C-N) of secondary aromatic amide.

### 3.4. Synthesis of N-(4-(Methylthio)phenyl)acetamide (***4***)

To compound **3** (0.33 g, 2 mmol) in distilled water (20 mL), triethylamine (0.30 mL, 2.2 mmol) were added and the mixture stirred for 10 min at room temperature, and then brought to 0 °C in an ice bath and CH_3_I (0.12 mL, 2 mmol) was added dropwise with vigorous stirring for 3 h. The product (thioether) was isolated by filtration without further purification as a pale yellow powder [29] (64% yield); m.p. 123–125 °C (lit. [as stated by the manufacturer, Chemical Trading Guidechem] 128 °C). R_f_ = 0.75. IR (cm^−1^): 3,282 (N-H) of secondary amide, 1,654 (C=O) of secondary amide, 1,599, 1,537 and 1,494 (aromatic), 1,319 (C-N) of secondary aromatic amide.

### 3.5. Synthesis of N-(4-(Methylsulfonyl)phenyl)acetamide (***5***)

Compound **4** (0.18 g, 1 mmol) was dissolved in 95% ethanol (30 mL), and 30% H_2_O_2_ (0.22 mL, 2 mmol) was added with continuous stirring for 1 h at room temperature, and then the solvent was evaporated to dryness to give compound **5** as an off-white powder, which was used without further purification [30] (68% yield); m.p. 190–192 °C. R_f_ = 0.65. IR (cm^−1^): 3,354 (N-H) of secondary amide, 1,687 (C=O) of secondary amide, 1,589, 1,535 and 1,502 (aromatic), 1,365 and 1,138 (O=S=O) of sulfone.

### 3.6. Synthesis of 4-(Methylsulfonyl)aniline (***6***)

Compound **5** (2.13 g, 10 mmol) was transferred to a flask containing a mixture of concentrated hydrochloric acid (10 mL) and water (30 mL). The mixture was gently boiled under reflux for 90 min, cooled to room temperature and activated charcoal (2 mg) was added. The mixture was heated to boiling and filtered with suction through a hardened filter paper. The filtrate was placed in a beaker and sodium bicarbonate was added in portions with stirring until the suspension become neutral (litmus paper). The mixture was cooled in ice bath and filtered by suction and dried to give compound **6** as a white powder [30] (44% yield); m.p. 132–134 °C (lit. [31] 131–135 °C); R_f_ = 0.78; IR (cm^−1^): 3,479 and 3,375 (N-H) of primary amine, 1,595 and 1,502 (aromatic), 1,294 (C-N) of primary amine, 1,313 and 1,145 (O=S=O) of sulfone.

### 3.7. General Procedure for Synthesis of Acid Anhydride Derivatives of NSAIDs ***7-10***

The anhydrides intermediates **7**–**10** were was obtained when two moles of carboxylic acid- containing compound were dissolved in tetrahydrofuran (THF, 30 mL), and then one mole of dicyclohexyl carbodiimide (DCC) was added. The reaction mixture was continuously stirred at room temperature for 4 hours, whereby a white precipitate of dicyclohexylurea (DCU) was formed, which then removed by filtration. The solvent was evaporated under vacuum to yield anhydrides **7**–**10** [32].

*2-(6-Methoxynaphthalene-2-yl)propanoic anhydride* (**7**): white powder (75% yield); m.p. 128–130 °C; R_f_ = 0.45. IR (cm^−1^): 1,801 and 1,737 of anhydride (symmetric and asymmetric), 1,606 and 1,471 (aromatic), 1,313, 1,224, 1,159 C-(C=O)-O-(C=O)-C of anhydride.

*2-(1-(4-Chlorobenzoyl)-5-methoxy-2-methyl-1*H*-indol-3-yl)acetic anhydride* (**8**): pale yellow powder (67% yield); m.p. 149–151 °C. R_f_ = 0.66. IR 1,807 and 1,727 of anhydride (symmetric and asymmetric), 1,604 & 1,496 (Aromatic), 1,287, 1,224, 1,166 C-(C=O)-O-(C=O)-C of anhydride.

*2-(2-(2,6-Dichlorophenylamino)phenyl)acetic anhydride* (**9**): white powder (62.3% yield); m.p. 107–110 °C; R_f_ = 0.77; IR (cm^−1^): 1,807 and 1,728 of anhydride (symmetric and asymmetric), 1,604, 1,504 and 1,454 (aromatic), 1,394, 1,265, 1,176 C-(C=O)-O-(C=O)-C of anhydride.

*3-(2,3-Dimethylphenylamino)benzoic anhydride* (**10**): yellow powder (65% yield); m.p. 164–166 °C; R_f_ = 0.68. IR (cm^−1^): 1,828 and 1,720 of anhydride (symmetric and asymmetric), 1,658, 1,510 and 1,456 (aromatic), 1,327, 1,228, 1,178 C-(C=O)-O-(C=O)-C of anhydride.

### 3.8. General Procedure for Synthesis of the Final Compounds ***11–14***

A mixture of the appropriate compound **7**–**10** (6 mmol), intermediate **6** (2.06 mg, 12 mmol), zinc dust (0.011 g), glacial acetic acid (1.1 mL, 19.2 mmol) and dioxanw (35 mL) were placed in a flask equipped with reflux condenser, and boiling stones were added. The reaction mixture was refluxed gently for 90 min, the solvent was evaporated under vacuum, the residue was dissolved in ethyl acetate, washed with NaHCO_3_ (10%, 3×), HCl (1 N, 3×), and distilled water (3×), and filtered over anhydrous magnesium sulfate. The filtrate was evaporated under vacuum to give the final compounds **11**–**14**. The final products were obtained as solids and the recrystallization was carried out by dissolving the compound in ethyl acetate and addition of petroleum ether (80–100 °C) to the filtrate until turbidity occurred and then keeping in a cold place overnight. The mixtures were filtered while cold and the precipitate was collected to give the final compounds **11**–**14** [33].

*2-(6-Methoxynaphthalen-2-yl)-*N*-(4methylsulfonyl)phenyl)propanamide* (**11**): pale yellow powder (46% yield); m.p. 228–230 °C; R_f_ = 0.68; IR (cm^−1^): 3,298 (N-H) of secondary amide, 1,658 (C=O) of secondary amide, 1,597 and 1,518 (aromatic), 1,336 and 1,165 (O=S=O) of sulfone, 1,257 (C-O-O) of aryl alkyl ether; CHNS calculated (C_21_H_21_NO_4_S): C, 65.78; H, 5.52; N, 3.65; S, 8.36; found: C, 63.9; H, 5.25; N, 3.81; S, 8.14.

*2-(1-(4-Chlorobanzoyl)-5-methoxy-2-methyl-1*H*-indol-3-yl)-N-(4-(methylsulfonyl)phenyl)acetamide* (**12**): pale yellow powder (46% yield); m.p. 228–230 °C; R_f_ = 0.68. IR (cm^−1^): 3,321 (N-H) of secondary amide, 1,641 (C=O) of secondary amide, 1,577, 1,508 and 1,450 (aromatic), 1,319 and 1,120 (O=S=O) of sulfone; CHNS calculated (C_26_H_23_ClN_2_O_5_S): C, 61.11; H, 4.54; N, 5.48; S, 6.28; found: C, 62.19; H, 4.72; N, 5.57; S, 6.54.

*2-(2-(2,6-Dichlorophenylamino)phenyl)-*N*-(4-(methylsulfonyl)phenyl)acetamide *(**13**): pale yellow powder (46% yield); m.p. 228–230 °C; R_f_ = 0.68; IR (cm^−1^): 3,474 (N-H) of secondary amide, 1,693 (C=O) of secondary amide, 1,572, 1,506, 1,454 (aromatic), 1,301 and 1,159 (O=S=O) of sulfone; CHNS calculated (C_21_H_18_Cl_2_N_2_O_3_S): C, 56.13; H, 4.04; N, 6.23; S, 7.14; found: C, 58.78; H, 4.17; N, 6.32; S, 6.92.

*3-(2,3-dimethylphenylamino)-*N*-(4-(methylsulfonyl)phenyl)benzamide *(**14**): pale yellow powder (46% yield); m.p. 228–230 °C; R_f_ = 0.68; IR (cm^−1^): 3,354 (N-H) of secondary amide, 1,670 (C=O) of secondary amide, 1,595, 1,529, 1,444 (aromatic), 1,311 and 1,169 (O=S=O) of sulfone; CHNS calculated (C_22_H_22_N_2_O_3_S): C, 66.98; H, 5.62; N, 7.10; S, 8.13; found: C, 65.23; H, 5.49; N, 7.46; S, 8.39.

### 3.9. Pharmacology

Albino rats of either sex weighing (150 ± 10 g) were supplied by the animal house of the College of Pharmacy, University of Baghdad, and were housed in the same location under standardized conditions. Animals were fed commercial chaw and had free access to water ad *libitum*. Animals were divided into six groups (each group consisting of six rats) as follows:

*Group A*: Six rats that served as control; and treated with the vehicle (propylene glycol 50% v/v). 

*Group B*: Six rats treated with diclofenac sodium as reference substance in a dose of 3 mg/kg [34], suspended in propylene glycol 50% (v/v).

*Group C*–*f*: Six rats/group treated with the tested compounds **11**–**14**, respectively, in the doses that determined below (suspended in propylene glycol 50% v/v) equivalent to 3 mg/kg of diclofenac sodium as a finely homogenized suspension in 50% v/v propylene glycol in water.

### 3.10. Anti-Inflammatory Activity

The anti-inflammatory activity of the tested compounds was studied using the egg-white induced edema model [33]. Acute inflammation was produced by a subcutaneous injection of undiluted egg-white (0.05 mL) into the plantar side of the left hind paw of the rats; 30 min after i.p. administration of the drugs or their vehicle. The paw thickness was measured by vernea at seven time intervals (0, 30, 60, 120, 180, 240, and 300 min) after drug administration.

The data was expressed as the mean ± SEM and results were analyzed for statistical significance using student t-test (Two Sample Assuming Equal Variances) for comparison between mean values. While comparisons between different groups were made using ANOVA: Two factors without Replication. Probability (P) value of less than 0.05 was considered significant.

## 4. Conclusions

An *in vivo* anti-inflammatory study showed that the incorporation of 4-(methylsulfonyl)aniline into well known NSAIDs (naproxen, indomethacine, diclofenac and mefanamic acid) maintained or increase the anti-inflammatory activity. Compounds **12** and **13** showed a comparable effect to that of diclofenac sodium, while compounds **11** and **14** might show higher effects comparable to that of diclofenac sodium.
